# Sedation versus General Anesthesia for Cardiac Catheterization in Infants: A Retrospective, Monocentric, Cohort Evaluation

**DOI:** 10.3390/jcm10235648

**Published:** 2021-11-30

**Authors:** Marian Mikus, Thomas Welchowski, Ehrenfried Schindler, Martin Schneider, Nathalie Mini, Mathieu Vergnat

**Affiliations:** 1 Department of Anaesthesiology and Intensive Care Medicine, University Hospital Bonn, 53127 Bonn, Germany; marian.mikus@ukbonn.de; 2 Institute of Medical Biometry, Informatics and Epidemiology, Medical Faculty, University of Bonn, 53127 Bonn, Germany; welchow@imbie.uni-bonn.de; 3 Department of Cardiology, German Paediatric Heart Centre, University Hospital of Bonn, 53127 Bonn, Germany; martin.schneider@ukbonn.de (M.S.); nathalie.mini@ukbonn.de (N.M.); 4 Department of Paediatric Cardiac Surgery, German Paediatric Heart Centre, University Hospital of Bonn, 53127 Bonn, Germany; mathieu.vergnat@ukbonn.de

**Keywords:** catheterization, infants, sedation, generalized additive models, inverse probability treatment weighting, stabilized weights

## Abstract

Background: Children with congenital heart disease require repeated catheterization. Anesthetic management influences the procedure and may influence outcome; however, data and recommendations are lacking for infants. We studied the influence of sedation versus general anesthesia (GA) on adverse events during catheterization for children <2 years old. Methods: We conducted a monocentric, retrospective study of all catheterization procedures (2008–2013). High-severity adverse event (HSAE) rates were compared using propensity-score-adjusted models, including pre- and intra-procedural variables. Results: 803 cases (619 patients) (368 (46%) GA, 435 (54%) sedation) with a mean age of 6.9 ± 6.1 months were studied. The conversion rate (GA after sedation) was 18 (4%). Hospital stay was 4.9 ± 4.0 and 4.1 ± 2.5 (*p* = 0.01) after GA or sedation, respectively. HSAE occurred in 75 (20%) versus 40 (9%) (*p* < 0.01) in GA versus sedation procedures, respectively. Risk factors (multivariable analysis) were older patients (*p* = 0.05), smaller weights (*p* < 0.01), palliated status (OR 3.2 [1.2–8.9], *p* = 0.02), two-ventricle physiology (OR 7.3 [2.7–20.2], *p* < 0.01), cyanosis (OR 4.6 [2.2–9.8], *p* < 0.01), pulmonary hypertension (OR 5.6 [2.0–15.5], *p* < 0.01), interventional catheterization (OR 1.8 [1.1–3.2], *p* = 0.02) and procedure-type risk category 4 (OR 28.9 [1.8–455.1], *p* = 0.02). Sedation did not increase the events rate and decreased the requirement for hemodynamic support (OR 5.2 [2.2–12.0], *p* < 0.01). Conclusion: Sedation versus GA for cardiac catheterization in children <2 years old is safe and effective with regard to HSAE. Sedation also decreases the requirement for hemodynamic support. Paradoxical effects (older age and two-ventricle physiology) on risk have been found for this specific age cluster.

## 1. Introduction

Children with congenital heart disease require repeated catheterization procedures to assess disease progression and therapeutic options [1]. Anesthetic management in the catheterization laboratory is an integral part of the process and significantly impacts the quality of the procedure, patient safety and comfort [2]. Both sedation and general anesthesia (GA) are frequently used but directly influence cardiopulmonary physiology [3]. The American Society for Anesthesiologists, the American Academy of Pediatrics and the German Society for Pediatric Cardiology released guidelines for sedation outside of the operating room in pediatric patients [4]. For infants, there are no strong recommendations. The literature shows the use of GA in up to 90% of neonates and up to 86% of children under 1 year of age [2].

Since 2008, our center has adopted a deliberate sedation policy for all pediatric catheterizations except for those requiring transesophageal echocardiography (TOE).

The goal of this study was to determine the impact of anesthetic management on the adverse events rate during cardiac catheterization in young children.

## 2. Materials and Methods

### 2.1. Patient Population

We performed a single-center retrospective cohort study. The study was approved by the Ethics Committee of the Medical Faculty, Rheinische Friedrich-Wilhelms Universität Bonn (IRB Number 455/19). Institutional Review Board approval was obtained to conduct this retrospective study and individual consent was waived.

All patients less than 2 years of age who underwent cardiac catheterization under sedation or GA at the German Heart Centre Sankt Augustin between 2008 and 2013 were included. Exclusion criteria were: emergency cases, requirement for TOE or surgery, surgery performed in the last 6 h.

Demographic variables recorded include age, weight, American Society of Anesthesiologists Physical Status, cardiac diagnoses, cyanosis (defined as transcutaneous O_2_ saturation < 90% during procedure), single or 2-ventricle physiology, pulmonary hypertension (PAH), surgical status (native (not operated), palliated (operated without 2-ventricle status), repaired (operated with 2-ventricle status)) and extracardiac anomalies (genetic, chromosomal or multi morbid syndrome, prematurity (<35 gestation weeks), enterocolitis).

### 2.2. Procedural Characteristics

Procedural variables included procedure type (diagnostic or interventional) and procedure-type risk category (based on the Congenital Cardiac Catheterization Project on Outcomes risk categories [3]).

Hemodynamic data included lowest systolic arterial pressure throughout the procedure, lowest mean arterial pressure throughout the procedure, left atrial pressure, biological data (base excess, lactate, systemic arterial blood saturation and central venous saturation) and need for intravenous medications (puffer requirement). The requirement for additional hemodynamic support (vasoactive and inotropic medications) was specifically recorded if hemodynamic support had to be initiated (patient with no support before the procedure) or intensified (patient already on support before the procedure) throughout the procedure.

### 2.3. Anesthetic Technique

All procedures were performed by a restrictive team of experienced staff members (2 cardiologists and 4 anesthesiologists, with more than 10 years of experience).

General anesthesia was achieved by inhalational or intravenous induction followed by intubation and controlled ventilation. Age-adjusted endotracheal microcuff tubes were used for airway control. Balanced anesthesia was maintained with a standardized protocol with sevoflurane (minimum alveolar concentration 0.5%), remifentanil (10–20 mcg/kg/h) and rocuronium (0.3 mg/kg for intubation).

Sedation protocol was achieved by a continuous intravenous infusion of propofol (5–10 mg/kg/h) and ketamine (0.5–1 mg/kg) while maintaining spontaneous ventilation under CO_2_ monitoring via nasal cannula.

Similar standard anesthetic monitoring was used for both techniques (non-invasive blood pressure, pulse rate, pre- and post-ductal transcutaneous O_2_ saturation, electrocardiogram (ECG), central and peripheral temperature, invasive blood pressure from catheterization, bispectral index).

**Figure 1:**
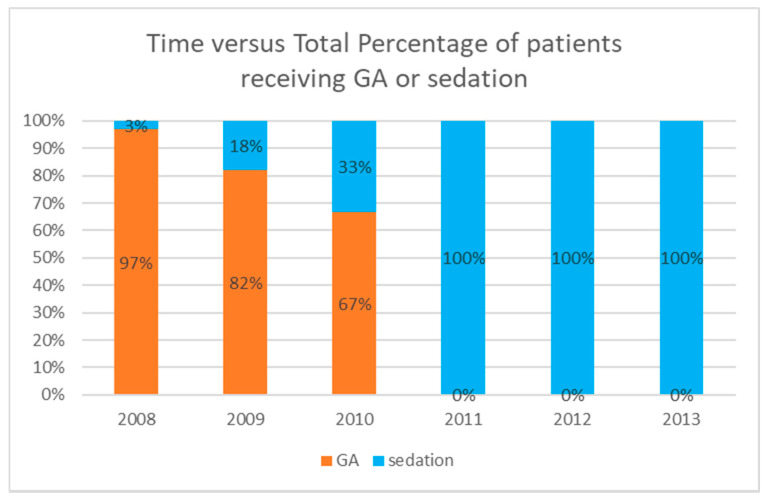
Chronological utilization trend between general anesthesia and sedation.

Over the years, patients were progressively assigned to cardiac catheterization from the GA group to the sedation group (Figure 1). Cases with initial sedation that were converted to GA because of an adverse event were included in the sedation cohort as an intention-to-treat analysis.

### 2.4. Early Outcome

24 h mortality, transfer to the intensive care unit (ICU), hospital length of stay and requirement for blood transfusion were recorded.

Adverse event severity was ranked according to a quantitative response five-level severity scale [3] and further grouped according to low (severity level 1—none/very mild and level 2—minor) and high (severity level 3—moderate, level 4—major, and level 5—catastrophic) similar to Lin [2] (Table 1).

To further emphasize the source of high-severity adverse events, the course of event for each high-severity adverse event patient was investigated and a qualitative explanatory classification was detailed: requirement for ICU monitoring, hypotension, respiratory failure, rhythm or conduction disturbance. Resuscitation events were also collected.

### 2.5. Statistical Analysis

Categorical variables were summarized with frequencies and percentages, and continuous variables with mean values and standard deviation. Comparisons between groups were made using the Chi-square test for categorical variables and the Student *t*-test for continuous variables. The continuous variable “time since beginning of experience” is defined as the time between the day of the procedure and the beginning of the study. The primary outcome of interest was the occurrence of high-severity adverse events. The secondary outcome was the requirement for additional hemodynamic support.

We applied generalized additive models [5] to the outcome of high-severity events (binomial distribution) on observational data to investigate the interventional effect of sedation versus GA. Covariates were chosen following medical relevance based on medical experience appraisal. The quantitative covariates age, weight, low systolic pressure and month since beginning experience were specified as thin-plate regression splines with maximal possible dimension of 10 [6]. Propensity score analysis based on inverse probability treatment (IPT) weighting was applied to better balance the sedation and GA groups regarding all other covariates and reduce the potential bias of nonrandomized treatment. The IPT model adjusts for all covariates specified in the final model. Following the recommendations given by Austin [7], we compared unstabilized and stabilized weight approaches regarding absolute standardized differences of each covariate between sedation and GA groups. On average, stabilized weights were chosen because these yielded on average smaller deviations across all covariates. To reduce alpha error inflation, we rescaled the IPT weights to sum up to the original sample size [8]. The statistical analysis of hemodynamics was performed analogously.

## 3. Results

### 3.1. Study Population

**Figure 2:**
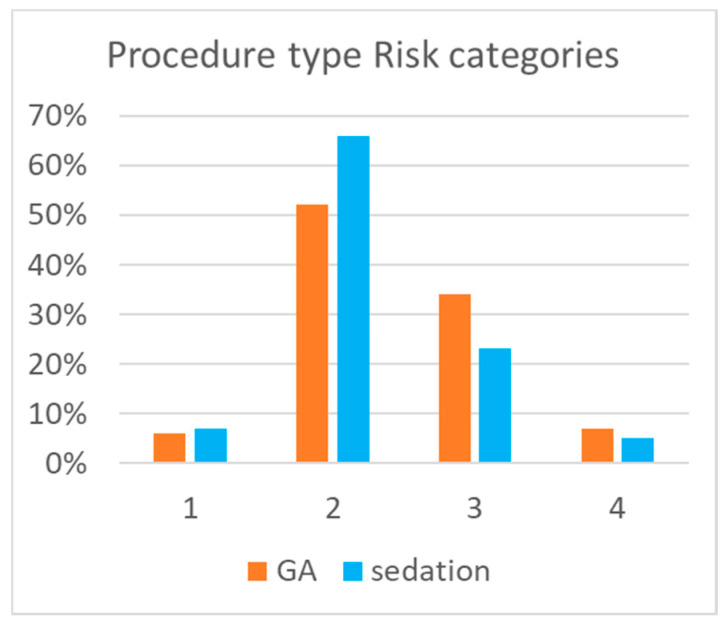
Distribution of procedure -type risk in each general anesthesia or sedation groups.

The study population included 803 cases in 619 patients. Of these, 435 (54%) were performed using sedation. Pre-procedural characteristics of patients in the GA and sedation cohorts are detailed in Table 2. The median weight of subjects was higher in the sedation cohort (*p* = 0.01). In addition to weight, cases with sedation were performed in subjects having a lower portion of higher (3–4) procedure-type risk categories (Figure 2). Catheter indications were balanced between diagnostics (357 (45%)) and intervention (446 (55%)).

### 3.2. Adverse Events

Procedural and post-procedural early characteristics are listed in Table 3.

Of the 453 cases conducted with sedation, 18 (4%) required conversion to intubation (10 apnea, 3 PAH crisis, 3 rhythm or conduction disturbance, 1 vessel rupture, 1 pulmonary hypoperfusion).

Adverse events were reported in 234 cases (29%), of which 115 (14%) events were categorized as high severity (category 3–5). Causes for high severity adverse events are summarized in Table 4. Rates of high severity for specific interventional procedures are listed in Table 5.

There were 17 (2%) major (category 4—life-threatening if not treated) adverse events.

Four (0.5%) catastrophic (category 5—resulting in death or extra-corporal membrane oxygenator (ECMO)) events occurred: one 8-day-old newborn with single-ventricle physiology (pulmonary atresia intact ventricle septum) under GA developed bradycardia during diagnostic catheterization and required resuscitation and ECMO support; two patients (one 2 months old with complex single-ventricle malformation and one 7 months old with cardiomyopathy) under sedation developed rhythm disturbance (atrioventricular block and ventricle tachycardia, respectively) during diagnostic catheterization and required resuscitation and ECMO support; one 17-month-old patient with complex cyanotic two-ventricle malformation under sedation suddenly died after the dilatation of major aorto-pulmonary collateral arteries.

There were two in-hospital deaths, both in the sedation group: one 7-month-old patient with cardiomyopathy who developed ventricle tachycardia during diagnostic catheterization and required resuscitation and ECMO support and could not be weaned off support; one 17-month-old patient with complex cyanotic two-ventricle malformation who suddenly died during the procedure after the dilatation of major aorto-pulmonary collateral arteries.

A total of three sedation cases (0.7%) were converted to GA: one 2-month-old patient with complex single-ventricle malformation who developed atrioventricular block during diagnostic catheterization and required resuscitation and ECMO support; one 2-month-old patient with persistent ductus arteriosus, PAH and Down syndrome who required intubation for PAH crisis during interventional catheterization for ductus arteriosus closure; one 3-month-old patient with palliated hypoplastic left heart syndrome who received oversedation in response to agitation and eventually had to be intubated because of several apnea during diagnostic catheterization.

### 3.3. Predictors of High-Severity Adverse Events

General anesthesia cases had a higher rate of high-severity adverse events (20%) than sedation cases (9%; *p* < 0.01) (Table 3).

The role of patient and procedural characteristics in high-severity adverse events (category 3, 4 and 5) was evaluated using univariate and multivariable analysis (Table 6).

In the multivariable model, palliated status (OR 3.2, 95% CI 1.2–8.9, *p* = 0.02), two-ventricle physiology (OR 7.3, 95% CI 2.7–20.2, *p* < 0.01), cyanosis (OR 4.6, 95% CI 2.2–9.8, *p* < 0.01), PAH (OR 5.6, 95% CI 2.0–15.5, *p* < 0.01), interventional catheterization (OR 1.8, 95% CI 1.1–3.2, *p* = 0.02) and procedure-type risk category 4 (OR 28.9, 95% CI 1.8–455.1, *p* = 0.02) were independent predictors of high-severity adverse events.

**Figure 3:**
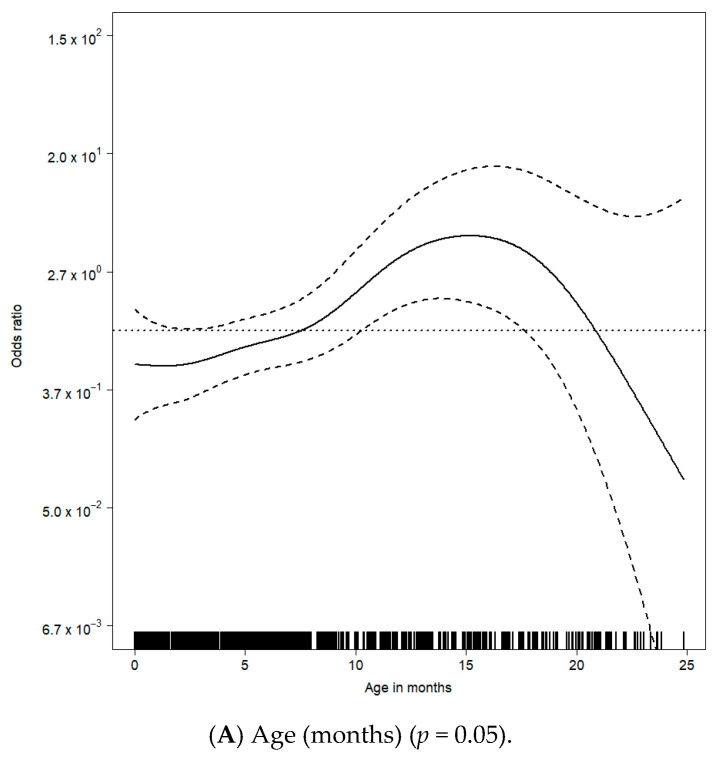

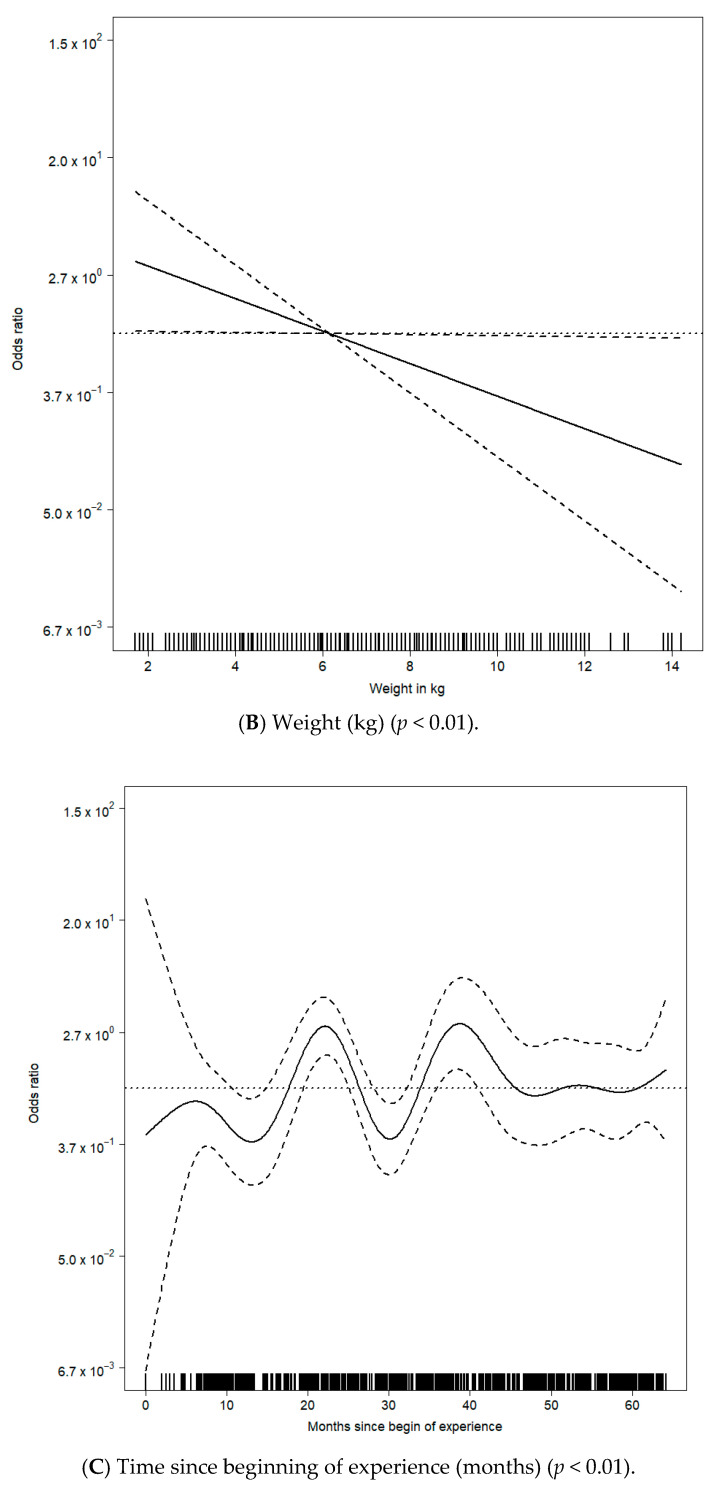

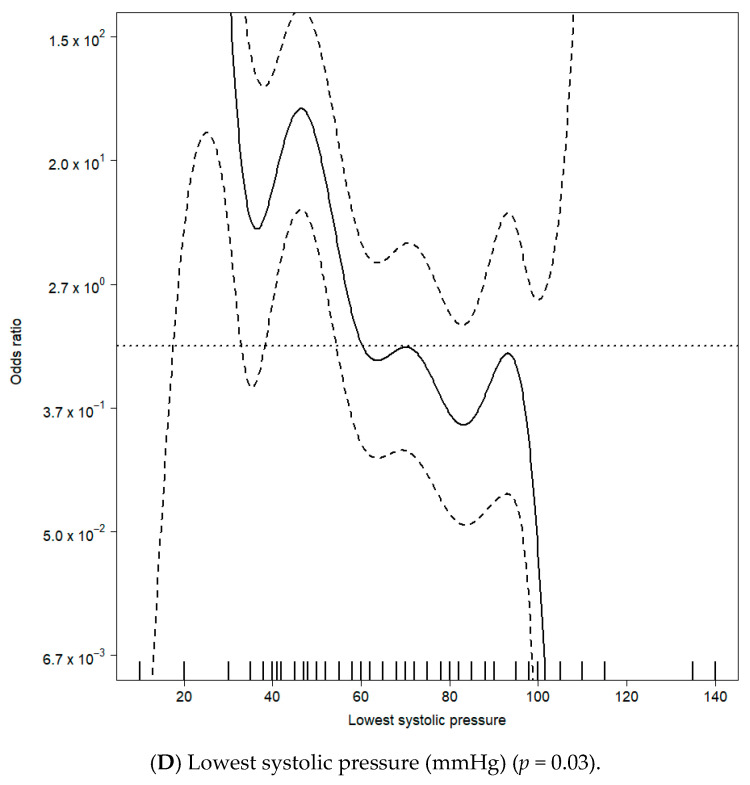
Multivariable association between high-severity adverse events and patient and procedural characteristics. (**A**) Age (months) (*p* = 0.05); (**B**) Weight (kg) (*p* < 0.01); (**C**) Time since beginning of experience (months) (*p* < 0.01); (**D**) Lowest systolic pressure (mmHg) (*p* = 0.03).

In a multivariable model using thin-plate regression splines with a maximal possible dimension of 10, we found that age (*p* = 0.05), weight (*p* < 0.01), time since beginning of experience (*p* < 0.01) and lowest systolic pressure (*p* = 0.03) were independently associated with high-severity adverse events (Figure 3).

A plot of odds ratios using the multivariable spline fit showed that an age of approximately 15.1 had the highest risk of high-severity adverse events (odds ratio of five) (Figure 3A), and it also showed a negative linear trend between weight and the risk of high-severity adverse events (Figure 3B), significant cyclic behavior of gathered experience with respect to the odds ratio of high severity around one (Figure 3C) and a negative linear trend between the lowest systolic pressure and the risk of high-severity adverse events (Figure 3D).

There was a multivariable association between high-severity adverse events and (A) age (months) (*p* = 0.05), (B) weight (kg) (*p* < 0.01), (C) time since beginning of experience (months) (*p* < 0.01) and (D) lowest systolic pressure (mmHg) (*p* = 0.03). Estimated high-severity adverse events odds ratio curves of continuous covariates were derived from the generalized additive model with thin-plate regression splines. For each observed value on the x-axis, small vertical lines are displayed on the bottom of each Figure 3A–D.

### 3.4. Predictors of Requirement for Additional Hemodynamic Support

General anesthesia cases had a higher rate of requirement for additional hemodynamic support (34%) than sedation cases (8%, *p* < 0.01) (Table 3).

The role of patient and procedural characteristics in the requirement for additional hemodynamic support was evaluated using univariate and multivariable analysis (Table 7).

In the multivariable model, the use of sedation (OR 0.1, 95% CI 0.1–0.2, *p* < 0.01), palliated status (OR 2.4, 95% CI 1.0–5.7, *p* = 0.05) and PAH (OR 7.1, 95% CI 3.0–16.9, *p* < 0.01) were independent predictors of the requirement for additional hemodynamic support.

**Figure 4:**
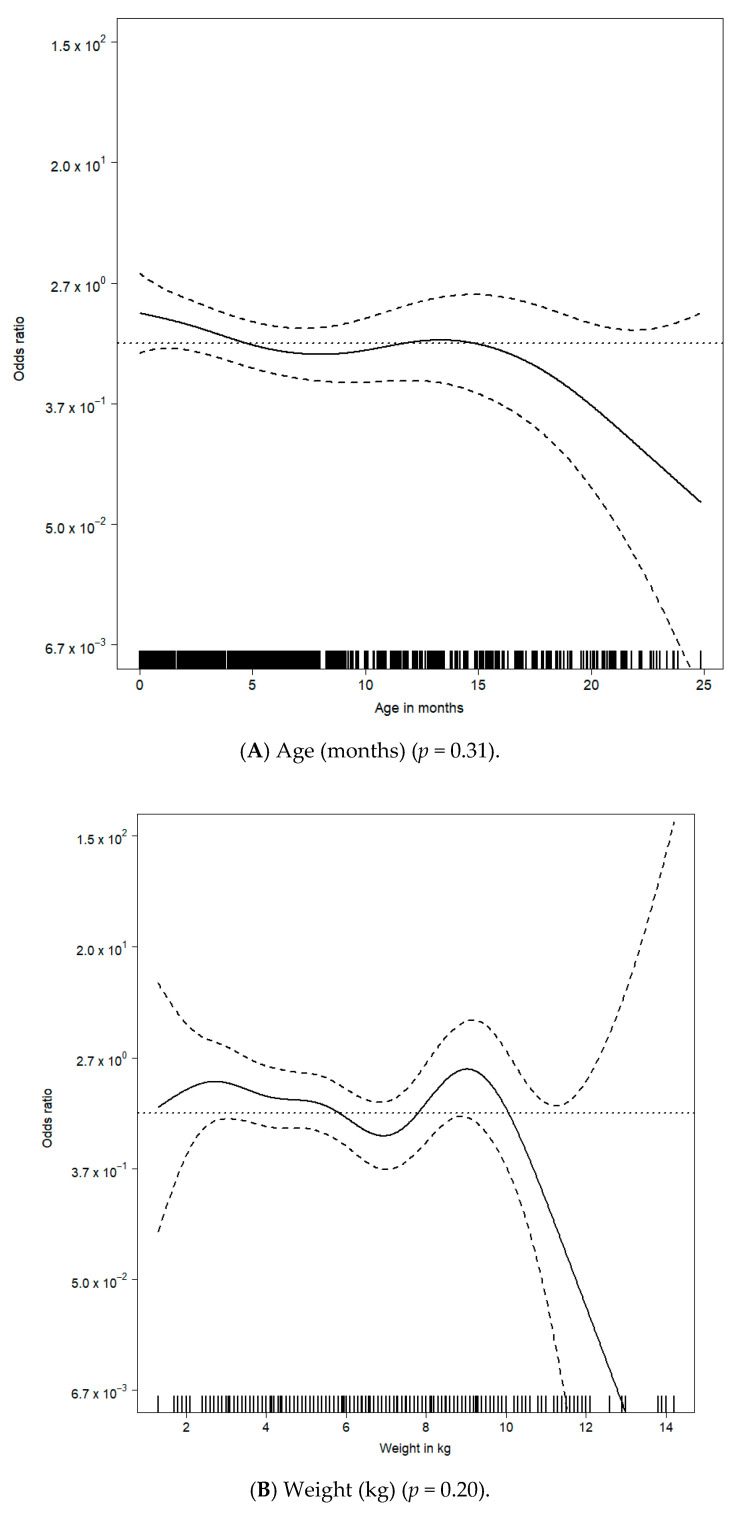

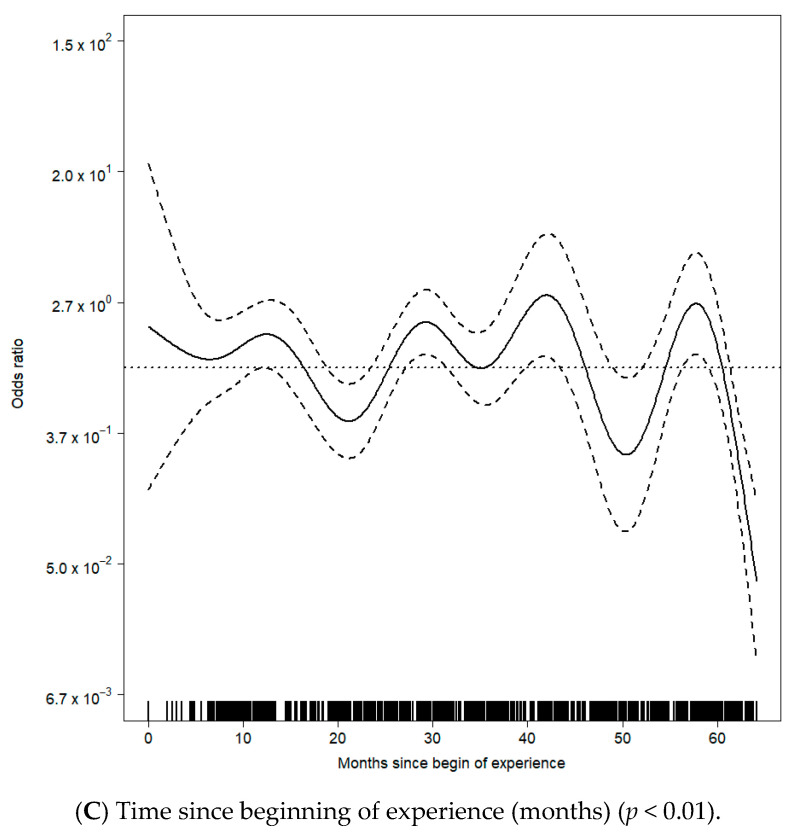
Multivariable association between requirement for additional hemodynamic support and patient and procedural characteristics. (**A**) Age (months) (*p* = 0.31); (**B**) Weight (kg) (*p* = 0.20); (**C**) Time since beginning of experience (months) (*p* < 0.01).

In a multivariable model using thin-plate regression splines with a maximal possible dimension of 10, we found that time since beginning of experience (*p* < 0.01) was independently associated with the requirement for additional hemodynamic support (Figure 4). A plot of odds ratios using the multivariable spline fit showed the significant cyclic behavior of gathered experience with respect to the odds ratio of high severity around one (Figure 4C).

There was a multivariable association between the requirement for additional hemodynamic support and (A) age (months) (*p* = 0.31), (B) weight (kg) (*p* = 0.21) and (C) time since beginning of experience (months) (*p* < 0.01).

Estimated additional hemodynamic support odds ratio curves of continuous covariates were derived from the generalized additive model with thin-plate regression splines.

For each observed value on the x-axis, small vertical lines are displayed on the bottom of each Figure 3A–C.

## 4. Discussion

In this single-center, retrospective study, we investigated the impact of anesthetic management on high-severity adverse events in children younger than 24 months during cardiac catheterization.

In an analysis adjusted for potential confounding factors, the risk of high-severity adverse events was not increased when using sedation instead of GA. A secondary analysis demonstrated that the use of sedation instead of GA significantly decreases the use of additional hemodynamic support.

### 4.1. Adverse Events

The data from the Impact [9] registry show rates of adverse events during cardiac catheterization from 31% and 30% for diagnostic versus interventional procedures, respectively, in neonates, to 26% and 21%, respectively, in infants (up to 1 year). Our reported rate (29% adverse event 2-3-4-5) fairly compares to such results. This reflects the complexity of cases and interventions performed in a very young population. Most serious events could be managed with CPR, surgery and ECMO, resulting in a limited 24-h mortality of 2 (<0.01%).

### 4.2. Sedation

The main finding of our study is that the use of sedation during cardiac catheterization in small children is safe, effective and non-inferior compared with GA, with regard to high-severity adverse events. Procedural sedation is a minimal mode of anesthesia, in which intubation is avoided, thus potentially reducing respiratory complications and hemodynamic disturbances (and potential vasopressor requirement) associated with mechanical ventilation. Another benefit of sedation is that it spares time, with an impact on cost and resources [10], but also reduces the requirement for the ICU with reduced hospital stays (as demonstrated in our data) and improved cost-effectiveness. A limitation for the sedation strategy is the requirement for TOE, which is unfeasible to plan with sedation in the child population.

Based on the results of the multivariable model, we can support the use of preprocedural risk stratification with procedure-type risk categories [3]. Patients and families with procedure risk type 4 should be informed and educated about the higher risk. We understand procedure risk type 4 as one of the independent predictors for high-severity adverse events. In such circumstances, the best preparation of rescue strategies (surgical stand-by, ECMO priming) is a crucial component for guaranteeing safety.

### 4.3. Weight

The higher incidence for adverse events in low-weight patient populations is not surprising and should be anticipated. The risk of adverse events in this group undergoing cardiac catheterization has been previously described [11].

### 4.4. Age Cluster

While there is a growing body of evidence in the literature that sedation can be used for children in the catheterization laboratory [2,10], sedation still remains underused—more dramatically in the specific small age cluster. Recent studies assessing sedation versus GA report a 9 and 14% (multicentric [2] and monocentric [10], respectively) sedation rate in patients who are less than 1 year old, while 31% and 32% of older patients were managed with sedation. We found that our 100% current rate of sedation (apart from emergency cases) in this small age (less than 2 years) cluster more deeply highlights the safety of this technique in the catheterization laboratory.

Additionally, this age group is also of specific interest as it is has been identified as a risk factor for adverse events [9,12]. Hemodynamic instability (uncorrected malformations, high-risk first-stage single-ventricle palliation (Norwood)) may restrain the spread of sedation techniques in these patients. In older patients, hemodynamics are more stable (already corrected malformation; more stable stage (Glenn, Fontan) for the single-ventricle patient), and thus the application of sedation techniques is more accepted and more widely used (31–32% of patients as aforementioned). Therefore, we sought to investigate the safety of sedation in a more demanding population where the use of this technique is less intuitive.

### 4.5. Age

Several studies have identified younger age as a risk factor for adverse events in children undergoing catheterization. Using the Congenital Heart Disease Adjustment for Risk Model over 8905 catheterization procedures from 2007 to 2010, the Congenital Cardiac Catheterization Outcomes Project identified age less than 1 year as a risk factor for adverse events [12]. As mentioned above, the Impact [9] registry showed the greatest adverse event rates during cardiac catheterization in neonates (31% and 30% for diagnostic versus interventional procedures, respectively), followed by infants (30 days to 1 year) (26% and 21%, respectively), whereas children (1 to 18 years) have a 5% and 7% risk, respectively. In our study focusing only on young (less than 2 years old) children, younger age was not associated with a higher rate of high-severity adverse events. Conversely, an age of 15 months was associated with a five times higher risk of high-severity adverse events. In younger patients, the rate of untouched (native) anatomy was higher (less than 1 month 96 (71%), 1 to 3 months 46 (40%), 3 to 6 months 68 (33%), 6 to 12 months 94 (51%), 12 to 24 months 73 (44%)). In our study, these patients with non-operated defects showed significantly more stable hemodynamics than operated patients and, more specifically, than operated patients without two-ventricle status (palliated). In our experience, these native patients show very stable hemodynamics through the catheterization procedure. Oppositely, older patients present in the catheterization laboratory with very complex anatomy and repaired and obviously remaining lesions that trigger indication for catheterization. They also obviously more often exhibit PAH after two-ventricle repair of complex obstructive lesions and more bronchial collateral circulation (source of hemoptysis) in response to chronic cyanosis. Younger patients do not exhibit these features. Post-surgical remaining lesions are also more complex (dilatation of previously placed stents or fibrotic vascular scars) to handle for catheter operators than native lesions. Therefore, older patients exhibit a higher risk of high-severity adverse events than younger infants.

Specific cardiac diagnoses have been identified as increasing risks for complications during cardiac catheterization: single ventricle and PAH.

### 4.6. Pulmonary Hypertension

Pulmonary hypertension is associated with an increased risk of perioperative cardiovascular complications [13,14]. Cardiac arrest and pulmonary hypertensive crises occur in 5.0% of the children undergoing cardiac catheterization [15]. Eight (21%) of our PAH patients experienced high-severity adverse events, and PAH was identified as an independent predictor for high-severity adverse events. Some mechanisms during catheterization (balloon catheter, dilatation) can trigger hemodynamic deterioration in patients with PAH. Pulmonary vascular resistance increase is a threatening condition for the catheter laboratory patient: these patients are almost impossible to resuscitate due to a lack of pulmonary blood flow secondary to increased pulmonary vascular resistance and pulmonary artery pressure, leading to a lack of venous return to the left heart and low cardiac output. In this patient population, it is important to maintain preload, potentially to start inotropic support prior to induction and to have inhaled nitric oxide available to prevent or treat a pulmonary hypertensive crisis or cardiac arrest.

The use of sedation may have a positive effect for such patients due to the better preservation of favorable hemodynamic parameters for the right ventricle (no modification of preload and afterload, as opposed to the use of GA with controlled ventilation). However, inadequate sedation can produce stress while oversedation can induce hypercarbia, hypoxemia and airway obstruction, with these having an impact on pulmonary vascular resistance, hemodynamic stability and measurements. Similar limitations can also occur with GA, which reduces right ventricle preload and increases its afterload. Some centers favor GA for the cardiac catheterization of PAH patients to avoid any PAH triggers [16].

### 4.7. Two-Ventricle

Unexpectedly, our multivariable analysis identified two-ventricle physiology as a risk factor for high-severity adverse events. Oppositely, using a risk model, the IMPACT registry identified single-ventricle physiology as critical for risk standardization [17]. The reason for such a striking result in our study may lie in the catheterization technique. Catheterization for a single ventricle at an early stage (before 2 years) is often straightforward: single aortography instead of selective coronarography; vessel occlusion of aortopulmonary collaterals; venous compartment direct connections with pulmonary arteries, easing diagnostics and intervention in these vessels. Oppositely, two-ventricle catheterization often requires a pathway through the heart chambers, making intervention and diagnosis more complex and also more prone to trigger rhythm or conduction disturbances. There is also the complex dilatation of fragile vessels (pulmonary atresia with major aortopulmonary collaterals) that can trigger PAH crisis or vascular tear.

### 4.8. Hemodynamics

Most anesthetic agents have significant hemodynamic effects, such as venodilation, decreased systemic vascular resistance and myocardial depression; positive pressure ventilation reduces venous return and preload and increases afterload on the right ventricle. Sedation provides normal intrathoracic pressure but can result in hypoventilation and hypercapnic acidosis if there is oversedation. However, our results showed a significant reduction in the requirement for additional hemodynamic support when patients were sedated.

There is a wide variability of anesthetic techniques used in the catheterization laboratory (various uses of sedation or GA, various professionals for sedation management [18], and lastly, various medications [19]). During cardiac catheterization in children, both anesthesiologists and cardiologists need to be aware of hemodynamic effects of anesthetic agents, as those can influence hemodynamic calculations that are crucial for decision-making for congenital heart diseases. Our choice of propofol and ketamine combination was motivated by the complementarity of these anesthetic agents. Propofol has known peripheral vasodilation effects. Ketamine increases the sympathetic tone and has an excellent safety profile in patients with pulmonary hypertension. In children with intracardiac shunting, excessive peripheral vasodilation (caused by propofol) increases right-to-left shunting and decreases the pulmonary to systemic blood flow ratio, which lead to arterial desaturation. Ketamine antagonizes the vasodilatory effects of propofol and also allows for propofol dosage reduction (which likewise decreases the occurrence of vasodilatory side effects of this agent).

After 10 years of experience, the current practice is a result of a slow and slight evolution of the initial concept. All patients are managed with the combination of propofol and dexmedetomidine. S-Ketamine is still used for blunting the stress response and pain reaction during venous or arterial access or during pulmonary balloon dilatation even when local anesthesia is used. We extended our standard anesthetic monitoring by using near-infrared spectroscopy (NIRS) in each patient.

### 4.9. Experience

The beginning of experience with sedation was motivated from the catheterization team and also other centers’ experience. At the beginning, the decision for GA or sedation was dictated by (i) clinical appraisal (severity of heart failure); (ii) the type of procedure (sedation initially chosen only for diagnostic catheterization).

To minimize complications, both GA and sedation were performed by a dedicated pediatric cardiac anesthesia team on a consultant level supported by dedicated trained nurses [3]. The results show initial slight fluctuations around 0 odds ratio to stabilization, with growing experience, to an absence of the effect of time on the rate of high-severity adverse events.

Whereas 31% of sedations for catheterization in Germany are performed without the presence of an anesthesiologist [18], in our study, only consultant-level anesthesiologists with experience in the management of children with congenital heart defects were involved, as defined in the guidelines of the German Society of Anesthesiology regarding the training of specialized pediatric cardiac anesthesiologists [20]. For each procedure, a ready-to-use anesthesia machine was present with all required drugs for rapid anesthesia induction as well as equipment for airway management. This allowed a very secure conversion from sedation to GA when required.

### 4.10. Limitations

This study is a single-center, observational, retrospective cohort analysis without randomization of treatment groups. We have compensated this by using the propensity score [7]. In addition, this study uses a self-reporting system and not all adverse events may have been exhaustively captured.

The increased use of sedation may also coincide with procedural improvements in catheterization that could bias the results.

In the study, some clusters had a low patient count (less than 10% of cohort: procedure-type risk categories 1 and 4, adverse severity levels ≥ 4, weight < 3 kg). Merged data of multi-center studies may increase the patient count for these low number categories and provide better appraisal of their influence on high-risk severity events than our study.

The adverse event severity 1 to 5 scale [3] was further grouped in low and high similar to Lin [2] and may appear oversimplified. The use of such dichotomous scaling nicely reflects the use of resources, as high severity is associated with the requirement for ICU. Furthermore, the use of a detailed 1 to 5 scale has added high complexity to the results and precluded any conclusion. Thus, similar to other authors [2], we chose a simplified and more pragmatic scaling system. This use also allows for comparison with other reports [2].

The study design was not planned to assess time. Several arguments plead against it: (i) the study includes a new experience (sedation), and thus the learning curve effect cannot be excluded; (ii) crude time analysis can be very misleading: multiple variables influence procedure duration, thus such a study requires a complete dedicated other multivariable analysis; (iii) from our experience, it seems that sedation may spare some time; however, wasted time lost from extubation in the GA group is not so noticeable as this extubation time superimposes itself with vascular access compression, so that the gained time with sedation is very discrete and needs a very large number of patients and thorough analysis; (iv) a surrogate for time could have been yearly patient number; however, again, such a parameter is also strongly influenced by multiple variables (center growth, team growth and renown, catheterization team learning curve, patient recruitment). Therefore, time was not collected in our data.

## 5. Conclusions

In this single-center, retrospective study of anesthetic management for cardiac catheterization in children younger than 24 months, the use of sedation was safe, effective and non-inferior compared with GA, with regard to high-severity adverse events. The use of sedation also resulted in a significant decrease in the requirement for additional hemodynamic support. In this specific cluster of young children, aside from the usual risk factors (smaller weight and pulmonary hypertension) for high-severity adverse events in cardiac catheterization, older age and two-ventricle physiology paradoxically increased the risk for high-severity adverse events. Further studies are required, at a multi-center level, to validate or refute our results and influence risk appraisal scores in this specific age cluster population.

## Figures and Tables

**Table 1:** Adverse event severity scale.

Severity Level		Definition
Low	1-None (very mild)	No harm, no change in condition, may have required monitoring to assess for potential change in condition with no intervention indicated.
	2-Minor	Transient change in condition, not life threatening, condition returns to baseline, required monitoring, required minor intervention such as holding a medication, or obtaining lab test.
High	3-Moderate	Transient change in condition may be life threatening if not treated, condition returns to baseline, required monitoring, required intervention such as reversal agent, additional medication, transfer to the intensive care unit for monitoring, or moderate trans-catheter intervention to correct condition.
	4-Major	Change in condition, life-threatening if not treated, change in condition may be permanent, may have required an intensive care unit admission or emergency readmission to hospital, may have required invasive monitoring, required interventions such as electrical cardioversion or unanticipated intubation or required major invasive procedures or trans-catheter interventions to correct condition.
	5-Catastrophic	Any death and emergency surgery or heart lung bypass support (ECMO) to prevent death with failure to wean from bypass support.

**Table 2:** Pre-procedural characteristics of patients.

	GA Procedures		Sedation Procedures		*p* Value
	(*n* = 368)		(*n* = 435)		
Mean age (m)	6.5 ± 6.0		7.3 ± 6.2		0.07
Mean weight (kg)	5.9 ± 2.5		6.3 ± 2.5		0.01
ASA physical status					0.00
1	0	(0%)	0	(0%)	
2	0	(0%)	0	(0%)	
3	38	(10%)	80	(18%)	
4	330	(90%)	355	(82%)	
Status					0.96
Native	172	47%	202	46%	
Palliated	132	36%	154	35%	
Repaired	64	17%	79	18%	
Single ventricle physiology	155 (42%)		178 (41%)		0.77
Cyanosis	207 (56%)		227 (52%)		0.25
Extracardiac anomalies	33 (9%)		52 (12%)		0.21
Pulmonary hypertension	16 (4%)		22 (5%)		0.74
Indication					0.70
Diagnostic	161 (44%)		196 (45%)		
Interventional	207 (56%)		239 (55%)		
Procedure type risk categories					<0.01
1	23	6%	30	7%	
2	193	52%	285	66%	
3	126	34%	99	23%	
4	26	7%	21	5%	

ASA: American Society of Anesthesiologists.

**Table 3:** Procedural and post-procedural early characteristics.

	GA Procedures		Sedation Procedures		*p* Value	Missing Values
	(*n* = 368)		(*n* = 435)			
Procedural						
Base excess	−2.6 ± 3.1		−3.7 ± 3		<0.01	7%
Puffer requirement	64 (17%)		32 (7%)		<0.01	0%
Lactate	1 ± 0.7		0.8 ± 1.1		0.01	17%
Blood saturation	85 ± 12		88 ± 10		<0.01	6%
Central venous saturation	57 ± 12		62 ± 11		<0.01	24%
Left atrial pressure	10 ± 4		9 ± 5		<0.01	45%
Lowest mean arterial pressure	47 ± 10		56 ± 11		<0.01	20%
Lowest systemic arterial pressure	60 ± 13		76 ± 15		<0.01	1%
Early post-procedural						
24 h mortality	0	0%	2	0.5%	0.99	
Intensive care admission	48	13%	27	6%	<0.01	
Transfusion	75	20%	55	13%	<0.01	
Hospital length of stay *	4.9 ± 4.0		4.1 ± 2.5		0.01	
Severity Level Adverse Event						
1	199	54%	369	85%	<0.01	
2	93	25%	26	6%		
3	65	18%	29	7%		
4	9	2%	8	2%		
5	1	0%	3	1%		
Low severity (level 1–2)	292	79%	395	91%	<0.01	
High severity (level 3–5)	75	20%	40	9%		
Requirement for additional hemodynamic support	127 (34%)		35 (8%)		<0.01	0%

* Only patients with simple course (442 (55%)): postoperative patients or patients requiring surgery were excluded.

**Table 4:** High-severity adverse event causes.

Cause	*n* = 115 (100%)
Requirement for ICU monitoring *	39 (34%)
Hypotension	31 (27%)
Respiratory failure	26 (23%)
Rhythm or conduction disturbance	19 (17%)
Resuscitation, independently of cause	16 (14%)

* Mixed causes of circulatory and/or respiratory failure, requirement for monitored therapy (fibrinolyse), residual effect of anesthesia (oversedation), threatening anatomic lesions, pulmonary hypertension crisis.

**Table 5:** Intervention in catheterization laboratory and high-severity adverse events.

	*n*	High-Severity Adverse Event, *n* (% [95%CI])
Pulmonary arteries (dilatation or stent) intervention	104	16 (15% [9–24%])
Aortic arch (dilatation or stent) intervention	91	6 (7% [2–14%])
Patent ductus arteriosus closure	63	3 (5% [1–13%])
Aortopulmonary collateral closure	53	6 (11% [4–23%])
Balloon valvotomy	40	10 (25% [13–41%])
Rashkind procedure	21	10 (48% [26–70%])
Shunt (Blalock or Sano) intervention	20	5 (25% [9–49%])
Patent ductus arteriosus stenting	15	3 (20% [4–48%])
Right ventricle outflow tract procedure	9	3 (33% [7–70%])
Pulmonary artery banding dilatation	7	3 (43% [10–82%])
Systemic veins (dilatation or stent) intervention	6	1 (17% [0–64%])
Pulmonary veins (dilatation or stent) intervention	5	1 (20% [0–72%])
Biopsy	4	2 (50% [7–93%])
Ventricle septal defect closure	3	0 (0% [NA])
Other (fenestration occlusion, paraprosthesis leak closure)	2	1 (50% [1–99%])

**Table 6:** Predictors of high-severity adverse events and multivariable analysis.

	Number	Univariate Analysis		Multivariable Analysis	
		High-Severity Adverse Events (%)	Odds Ratio (95% CI)	Odds Ratio (95% CI)	*p* Value
Use of sedation	435	40 (9.2%)	1.2 (0.8–1.8)	1.2 (0.7–2.2)	0.46
Status					
Native	374	67 (17.9%)	1.0	1.0	
Palliated	286	35 (12.2%)	3 (2.1–4.4)	3.2 (1.2–8.9)	0.02
Corrected	143	13 (9.1%)	0.5 (0.2–1)	0.5 (0.2–1.4)	0.20
Physiology					
Single-ventricle	333	44 (13.2%)	1.0	1.0	
Two-ventricle	470	71 (15.1%)	0.3 (0.2–0.5)	7.3 (2.7–20.2)	<0.01
Cyanosis	434	77 (17.7%)	5.5 (3.5–8.4)	4.6 (2.2–9.8)	<0.01
Extracardiac anomalies	85	13 (15.3%)	0.7 (0.4–1.6)	0.7 (0.3–1.8)	0.44
Pulmonary hypertension	38	8 (21.1%)	1.4 (0.5–3.5)	5.6 (2.0–15.5)	<0.01
Interventional catheterization	446	71 (15.9%)	3.4 (2.2–5.2)	1.8 (1.1–3.2)	0.02
Procedure-type risk category					
1	53	1 (1.9%)	1.0	1.0	
2	478	52 (10.9%)	17.1 (1.2–239.7)	10.6 (0.8–142.5)	0.08
3	225	48 (21.3%)	11.9 (0.8–170.7)	4.7 (0.3–67.0)	0.25
4	47	14 (29.8%)	33 (2.1–510.4)	28.9 (1.8–455.1)	0.02

**Table 7:** Predictors of requirement for additional hemodynamic support and multivariable analysis.

	Number	Univariate Analysis		Multivariable Analysis	
		Requirement for Additional Hemodynamic Support (%)	Odds Ratio (95% CI)	Odds Ratio (95% CI)	*p* Value
Use of sedation	435	35 (8.0%)	0.2 (0.1–0.2)	0.1 (0.1–0.2)	<0.01
Status					
Native	374	67 (17.9%)	1.0	1.0	
Palliated	286	70 (24.5%)	1.9 (1.3–2.7)	2.4 (1.0–5.7)	0.05
Corrected	143	25 (17.5%)	0.4 (0.2–0.8)	1.5 (0.7–3.0)	0.28
Physiology					
Single-ventricle	333	83 (24.9%)	1.0	1.0	
Two-ventricle	470	79 (16.8%)	0.5 (0.3–0.7)	1.4 (0.6–3.2)	0.48
Cyanosis	434	112 (25.8%)	3.1 (2–4.8)	1.7 (0.9–3.4)	0.11
Extracardiac anomalies	85	15 (16.6%)	1.1 (0.6–1.9)	1.7 (0.8–3.6)	0.15
Pulmonary hypertension	38	8 (21.1%)	3 (1.4–6.3)	7.1 (3.0–16.9)	<0.01
Interventional catheterization	446	85 (19.1%)	0.7 (0.5–1.1)	0.9 (0.5–1.4)	0.53
Procedure type risk category					
1	53	7 (13.2%)	1.0	1.0	
2	478	72 (15.2%)	23.3 (1.6–346.9)	1.6 (0.5–5.5)	0.48
3	225	66 (29.3%)	16.1 (1.1–244.6)	3.4 (0.9–12.9)	0.07
4	47	17 (36.2%)	46.5 (2.9–752.6)	4.5 (1.0–21.1)	0.06

## Data Availability

There are no supporting data.

## References

[1] Hollinger I., Mittnacht A., Lake C.L., Booker P.D. (2005). Cardiac Catheterisation and Other Radiographic Examinations. Pediatric Cardiac Anaesthesia.

[2] Lin C.H., Desai S., Nicolas R., Gauvreau K., Foerster S., Sharma A., Armsby L., Marshall A.C., Odegard K., DiNardo J. (2015). Sedation and Anaesthesia in Pediatric and Congenital Cardiac Catheterization: A Prospective Multicenter Experience. Pediatr. Cardiol..

[3] Bergersen L., Gauvreau K., Marshall A., Kreutzer J., Beekman R., Hirsch R., Foerster S., Balzer D., Vincent J., Hellenbrand W. (2011). Procedure-Type Risk Categories for Pediatric and Congenital Cardiac Catheterization. Circ. Cardiovasc. Interv..

[4] Cote C.J., Wilson S. (2019). Guidelines for monitoring and management of pediatric patients during and after sedation for diagnostic and therapeutic procedures: An update. Pediatrics.

[5] Wood S.N. (2017). Generalized Additive Models: An Introduction with R.

[6] Wood S. (2003). Thin plate regression splines. J. R. Stat. Soc. Ser. B Stat. Methodol..

[7] Austin P.C., Stuart E.A. (2015). Moving towards best practice when using inverse probability of treatment weighting (IPTW) using the propensity score to estimate causal treatment effects in observational studies. Stat. Med..

[8] Xu S., Ross C., Raebel M.A., Shetterly S., Blanchette C., Smith D. (2010). Use of Stabilized Inverse Propensity Scores as Weights to Directly Estimate Relative Risk and Its Confidence Intervals. Value Health.

[9] Vincent R.N., Moore J., Beekman R.H., Benson L., Bergersen L., Holzer R., Jayaram N., Jenkins K., Ringel R., Rome J. (2016). Procedural characteristics and adverse events in diagnostic and interventional catheterizations in paediatric and adult CHD: Initial report from the IMPACT Registry. Cardiol. Young.

[10] O’Byrne M.L., Millenson M.E., Steven J.M., Gillespie M.J., Dori Y., Glatz A.C., Rome J.J. (2019). Operator-Directed Procedural Sedation in the Congenital Cardiac Catheterization Laboratory. JACC Cardiovasc. Interv..

[11] Backes C.H., Cua C., Kreutzer J., Armsby L., El-Said H., Moore J.W., Gauvreau K., Bergersen L., Holzer R.J. (2013). Low weight as an independent risk factor for adverse events during cardiac catheterization of infants. Catheter. Cardiovasc. Interv..

[12] Lin C.H., Hegde S., Marshall A.C., Porras D., Gauvreau K., Balzer D.T., Beekman R.H., Torres A., Vincent J.A., Moore J.W. (2014). Risk and Management of Life threatening adverse events during cardiac catheterization for congenital heart disease. Pediatr. Cardiol..

[13] Taylor C.J., Derrick G., McEwan A., Haworth S.G., Sury M.R.J. (2007). Risk of cardiac catheterization under anaesthesia in children with pulmonary hypertension. Br. J. Anaesth..

[14] Van der Griend B.F., Lister N.A., McKenzie I.M., Martin N., Ragg P.G., Sheppard S.J., Davidson A.J. (2011). Postoperative Mortality in Children After 101,885 Anesthetics at a Tertiary Pediatric Hospital. Anesth. Analg..

[15] Carmosino M.J., Friesen R.H., Doran A., Ivy D.D. (2007). Perioperative Complications in Children with Pulmonary Hypertension Undergoing Noncardiac Surgery or Cardiac Catheterization. Anesth. Analg..

[16] Twite M.D., Friesen R.H. (2014). The Anesthetic Management of Children with Pulmonary Hypertension in the Cardiac Catheterization Laboratory. Anesthesiol. Clin..

[17] Jayaram N., Beekman R.H., Benson L., Holzer R., Jenkins K., Kennedy K.F., Martin G.R., Moore J.W., Ringel R., Rome J. (2015). Adjusting for Risk Associated with Pediatric and Congenital Cardiac Catheterization: A Report from the NCDR IMPACT Registry. Circulation.

[18] Bähner T., Heinze I., Dewald O., Mueller M., Schindler E., Schirmer U., Hoeft A., Baumgartner E., Ellerkmann R.K. (2016). Anästhesiologische Versorgung an deutschen Zentren für Kinderherzchirurgie. Aktueller Stand der personellen und strukturellen Organisation. Anästh. Intensivmed..

[19] Baehner T., Kiefer N., Ghamari S., Graeff I., Huett C., Pflugradt S., Sendzik B., Heinze I., Mueller M., Schindler E. (2020). A National Survey: Current Clinical Practice in Pediatric Anesthesia for Congenital Heart Surgery. World J. Pediatr. Congenit. Heart Surg..

[20] Schindler E., Koster A., Becke K. (2017). Personelle, räumliche, apparative und organisatorische Voraussetzungen sowie Anforderungen bei der Erbringung von Anästhesieleistungen für herzchirurgische und kardiologische Eingriffe bei Kindern und Jugendlichen mit angeborenen Herzfehlern. Anästh. Intensivmed..

